# Characterization of Kupffer cells in livers of developing mice

**DOI:** 10.1186/1476-5926-10-2

**Published:** 2011-07-12

**Authors:** Bryan G Lopez, Monica S Tsai, Janie L Baratta, Kenneth J Longmuir, Richard T Robertson

**Affiliations:** 1 Department of Anatomy & Neurobiology, School of Medicine, University of California, Irvine CA, USA; 2 Department of Physiology & Biophysics, School of Medicine, University of California, Irvine CA, USA; 3 Chao Family Cancer Center, University of California, Irvine CA, USA

## Abstract

**Background:**

Kupffer cells are well known macrophages of the liver, however, the developmental characteristics of Kupffer cells in mice are not well understood. To clarify this matter, the characteristics of Kupffer macrophages in normal developing mouse liver were studied using light microscopy and immunocytochemistry.

**Methods:**

Sections of liver tissue from early postnatal mice were prepared using immunocytochemical techniques. The Kupffer cells were identified by their immunoreactivity to the F4/80 antibody, whereas endothelial cells were labelled with the CD-34 antibody. In addition, Kupffer cells and endothelial cells were labelled by systemically injected fluorescently labelled latex microspheres. Tissue slices were examined by fluorescence microscopy.

**Results:**

Intravenous or intraperitonal injections of microspheres yielded similar patterns of liver cell labelling. The F4/80 positive Kupffer cells were labelled with both large (0.2 μm) and small (0.02 μm) diameter microspheres, while endothelial cells were labelled only with the smaller diameter microspheres. Microsphere labelling of Kupffer cells appeared stable for at least 6 weeks. Cells immunoreactive for F4/80 were identified as early as postnatal day 0, and these cells also displayed uptake of microspheres. Numbers of F4/80 Kupffer cells, relative to numbers of albumin positive hepatocytes, did not show a significant trend over the first 2 postnatal weeks.

**Conclusions:**

Kupffer cells of the developing mouse liver appear quite similar to those of other mammalian species, confirming that the mouse presents a useful animal model for studies of liver macrophage developmental structure and function.

## Background

The important roles performed by the liver in the storage and release of nutrients and in the neutralization and elimination of a variety of toxic substances have prompted investigations of its cellular constituents and organization. Some of these studies have been carried out in human liver, but the importance of having an experimental model system has prompted several investigations of liver organization in laboratory mammals, primarily rats [1-7]. In species studied thus far, investigations have demonstrated that the liver is comprised of parenchymal cells, the hepatocytes [8-10], and a variety of non-parenchymal resident cells including a population of macrophages termed Kupffer cells [1-3,6,7,11-15]. Kupffer cells form a partial lining of the liver sinusoids, acting to phagocytose foreign particulate matter from the circulating blood.

In recent years, the use of mice, and particularly genetically engineered mice, in research laboratories has increased markedly. Several studies have used mice in addressing questions of liver structure and function in general, and of Kupffer cells in particular [12-21]. Although several studies have examined varied aspects of Kupffer cell function in mice, there has not been, to our knowledge, a study of the basic characteristics and the postnatal development of Kupffer cells in mice. Because of the important role that will be played by mice in future studies of liver function, it is imperative to establish the baseline of normal Kupffer cell composition to serve as a reference for these future studies.

The purpose of this study was to identify and characterize Kupffer cells in the livers of postnatal mice, and to determine the age in mice at which Kupffer cells are phagocytically active.

## Results

### Immunocytochemical identification of Kupffer cells

**Figure 1:**
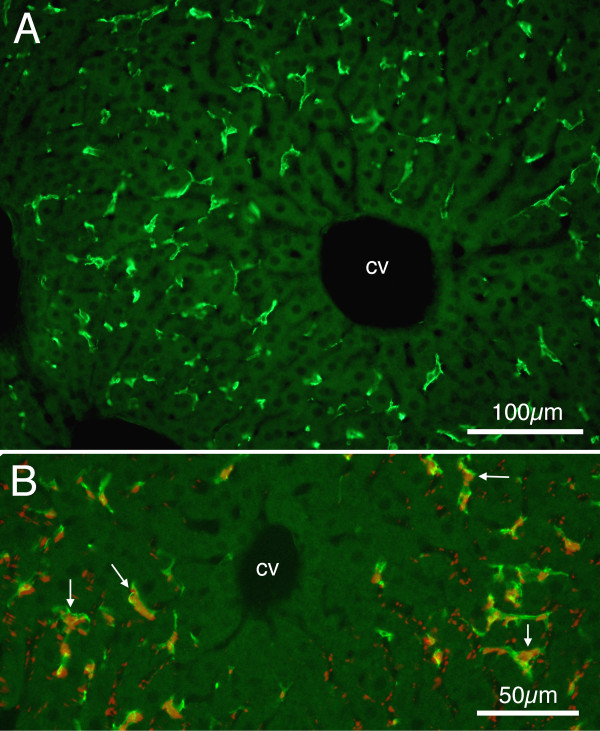
**Fluorescence photomicrographs showing Kupffer cells from sections of P28 mouse liver**. A: Alexa 488 (green) labelled F4/80 positive cells. Note branching of cells, and relative absence of positive cells close to the central venule (cv). Calibration bar = 100 μm. B: Merged image showing Alexa 488 (green) labelled F4/80 positive cells along with 0.2 μm red fluorescent microsphere positive cells. Arrows indicate examples of double labelled cells. Calibration bar = 50 μm.

The photomicrographs presented in Figure 1 are taken from mice euthanized at 28 days of age. These images demonstrate that at this relatively young age the F4/80 antibody labels a population of cells with widely branching and broad dendritic processes and apparently small oblong nuclei, quite similar to those reported for Kupffer cells in adults [12,21]. The F4/80 labelled cells are distributed rather homogeneously throughout the liver tissue, with the exception that these cells typically are not seen close to (within 50 μm of) the central venules.

Further, Figure 1B demonstrates that these F4/80 positive cells can be labelled by intravascularly administered fluorescent microspheres (in this case, 0.2 μm microspheres with a post-injection survival period of 1 hour), indicating their phagocytic ability. Although not all F4/80 positive cells can be seen to contain microspheres, and not all (red) microspheres can be seen to be contained within F4/80 positive cells, the correspondence of the two labels is remarkable. Greater than 90% of F4/80 positive cells contained microspheres.

### Size of microspheres

**Figure 2:**
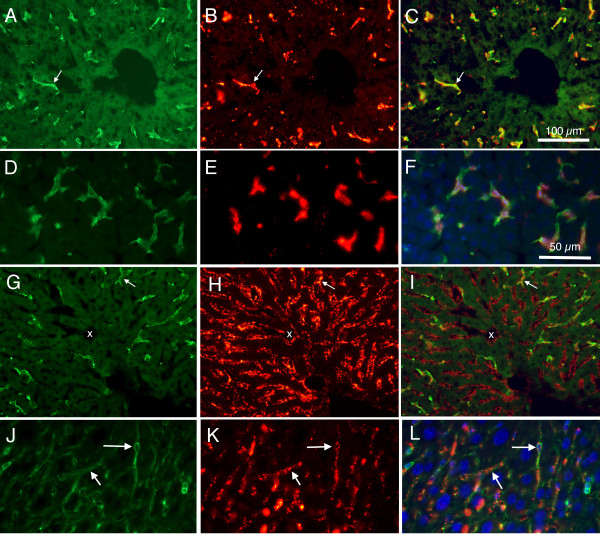
**Fluorescence photomicrographs from P30 and P15 mouse liver, showing difference in patterns of labeling between large (0.2 μm) and small (0.02) microspheres**. A: Alexa 488 labelled F4/80 cells from P30 mouse. B: Same section as in 'A' but viewed using rhodamine optics to reveal large (0.2 μm) fluorescently labelled microspheres. C: Merged image of 'A' and 'B', showing co-localization of F4/80 and large microspheres. D: Higher magnification photomicrograph showing Alexa 488 labelled F4/80 cells from P15 mouse liver. E: Same section as in 'D', viewed using rhodamine optics to reveal large (0.2 μm) fluorescently labelled microspheres. F: Merged image of 'D' and 'E', and also with ultraviolet imaging of DAPI labelled cell nuclei, showing cells co-labelled with F4/80 and microspheres. Note that most microspheres appear associated with F4/80 positive cells. G: Alexa 488 labelled F4/80 positive cells from P30 mouse. H: Same section as in 'G', viewed using rhodamine optics to reveal small (0.02 μm) fluorescently labelled microspheres. I: Merged image of 'G' and 'H', showing a few cells co-labelled with F4/80 and microspheres. Note that most microspheres appear not to be associated with F4/80 positive cells. White arrows indicate double labelled cells; x indicates capillary with red microspheres but absence of F4/80 immunoreactivity. J: Higher magnification photomicrograph showing Alexa 488 labelled CD-34 cells from P15 mouse liver. K: Same section as in 'J', viewed using rhodamine optics to reveal small (0.02 μm) fluorescently labelled microspheres. L: Merged image of 'J' and 'K', and also with ultraviolet imaging of DAPI labelled cell nuclei, showing cells co-labelled with CD-34 and microspheres. Note that most microspheres appear associated with CD-34 positive cells; examples are indicated by white arrows. Calibration bar in 'C' = 100 μm for images A, B, C, G, H, and I. Calibration bar in 'F' = 50 μm for images D, E, F, J, K, and L.

The pattern of labelling within the liver was influenced by the size of microspheres. For example, when mice were injected intravascularly with the relatively large 0.2 μm microspheres, these microspheres were found co-localized primarily with F4/80 positive cells. The regional distribution of these co-labelled cells from a P30 mouse is illustrated in Figure 2A,B,C. Images taken at higher magnification, and from younger P15 mice, in Figure 2D,E,F demonstrate morphological features of these cells. The morphological features of these cells correspond to Kupffer cells of mature liver.

In contrast, when the relatively smaller (0.02 μm) microspheres were injected intravascularly, they were found virtually continuously in the lining of the sinusoidal capillaries of the liver (Figure 2G,H,I). Some of these smaller microspheres were found within F4/80 labelled cells, but as shown in higher magnification of tissues from P15 mice, most of the smaller microspheres were found co-localized with the CD-34 antibody, specific for endothelial cells (Figure 2J,K,L).

### Temporal patterns of microsphere labeling

**Figure 3:**
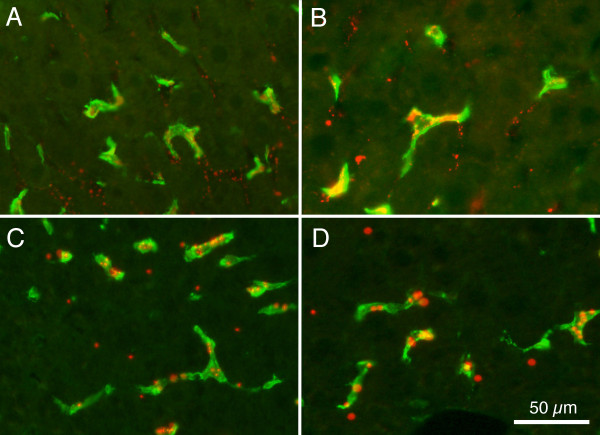
**Merged images of fluorescence photomicrographs from animals injected intravenously at P20 show Alexa 488 (green) labelled and large (0.2 μm) red fluorescent microsphere containing cells**. A: 30 minutes following IV injection. B: 1 hr following injection. C: 1 week following injection. D: 2 weeks following injection. Calibration bar in 'D' = 50 μm for all images.

Mice aged P20 were injected intravascularly with the larger (0.2 μm) microspheres and then allowed survival times ranging from 15 minutes to 6 weeks. Very few microspheres were detected in liver at the survival time of 15 minutes. Within 30 minutes, microspheres could be detected within F4/80 positive cells, but some microspheres also were found along the sinusoidal capillary walls without being clearly associated with F4/80 cells (Figure 3A). One hr following injection, F4/80 positive cells were clearly labelled with the microspheres (Figure 3B). Figures 3C and 3D show examples of labelling 1 week and 2 weeks respectively; these both resemble the material at 1 hour survival. At survival times of 2 weeks or longer (Figure 3D), the fluorescent microspheres appeared somewhat larger than at shorter times, possibly indicating the microspheres were being sequestered together in phagosomes. Microspheres could be detected at survival times of 6 weeks, the longest time investigated in this study.

### Comparison of IP and IV injections

**Figure 4:**
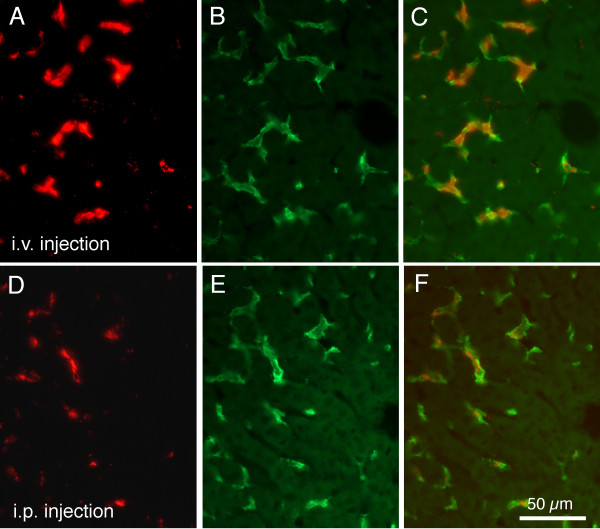
**Fluorescence images allow comparison of results of IV and IP injections**. Fluorescence images under rhodamine optics show labelling of mouse liver 1 hr following intravenous (A) or intraperitoneal (D) injections of red labelled large (0.2 μm) microspheres. The same sections were photographed under fluorescein optics (B and E) to show F4/80 immunoreactivity. Merged images in C and F demonstrate co-localization of red microspheres and green immunoreactivity. Calibration bar in F = 50 μm for all images.

One of the goals of this study was to determine the age at which Kupffer cells would show phagocytosis of fluorescent microspheres. Intravenous injections in younger mouse pups are challenging, so the efficacy of intraperitonal (IP) injections was explored. Figure 4 compares microsphere labeling of liver cells from age matched animals, both injected with the larger 0.2 μm microspheres at P16. One received an intravenous (IV) tail vein injection of fluorescent microspheres (Figure 4A,B,C) and the other (Figure 4D,E,F) receiving an IP injection. Both animals were euthanized 1 hour after the injection. The two injection procedures resulted in very similar distributions of labelling within the liver, with evidence of red fluorescent microspheres within green F4/80 immunoreactive cells in both cases (Figure 4C,F). Although the distributions of the fluorescently labelled microspheres in the two experimental paradigms were virtually identical, the IV injections typically yielded more intense labelling (compare Figure 4A and 4D). Because the present study was not intended as a quantitative assessment of phagocytic uptake of markers but rather a study of cell types that accumulate the microspheres, these data were interpreted to indicate that an IP injection could be used with confidence when conducting experiments on the small early postnatal mice.

### Development of microsphere labeling of Kupffer cells

**Figure 5:**
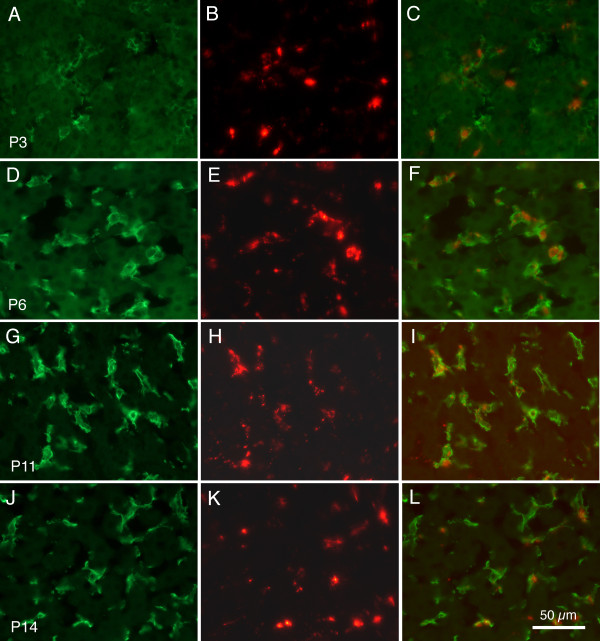
**Kupffer cells in developing mouse liver**. Fluorescence images showing Alexa 488 (green) F4/80 immunoreactivity and large 0.2 μm microspheres (red) labelling of cells in developing mouse liver. The left column (A, D, G J) presents F4/80 immunoreactivity. The middle column (B, E, H, K) presents microsphere fluorescence in the same sections as shown in A, D, and G. The right column (C, F, I, L) presents merged images from the left and middle columns. Top row, tissue from pup euthanized at P3; second row from P6, third row from P11, and bottom row from P14. Calibration bar in L = 50 μm for all images.

Figure 5 presents examples of microsphere labelling and F4/80 immunoreactivity in young mouse pups, following intraperitoneal injection of the larger (0.2 μm diameter) microspheres. Figure 5A,B,C demonstrate that in pups as young as P3, F4/80 positive cells could be detected, and many of these cells appear to contain the injected microspheres. The F4/80 positive cells displayed polygonal cell bodies, with ovoid nuclei, and appeared to have somewhat truncated processes. Figure 5D,E,F demonstrate that at P6, the F4/80 positive cells also appeared with polygonal cell bodies, ovoid nuclei, but with dendritic processes that appeared longer and wider than those seen from animals euthanized at P3. At P11 (Figure 5G,H,I) and at P14 (Figure 5J,K,L) the F4/80 positive cells appeared with more extensive dendritic branching; these patterns appear similar to those encountered in mature animals, as presented previously [21]. Immunoreactivity of the F4/80 antibody was present in every mouse examined; the general distribution of Kupffer cells did not display differences in mice aged from 3 days to 12 weeks.

### Relative numbers of Kupffer cells in developing mouse liver

The numbers of labelled Kupffer cells were studied in sections of livers taken from developing mice. Neighboring sections through liver were collected and processed for either F4/80 immunoreactivity or albumin immunoreactivity. Thus, numbers of F4/80 labelled Kupffer cells (with DAPI labelled nuclei) could be compared to numbers of albumin labelled hepatocytes (with DAPI labelled nuclei) in slices of similar thickness and from similar regions.

**Figure 6:**
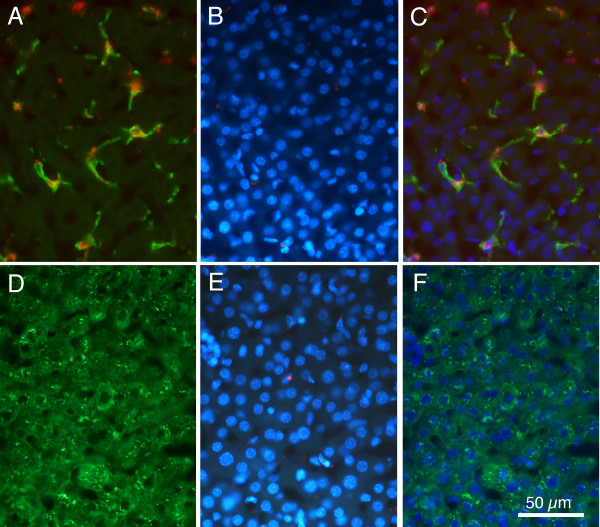
**Fluorescence images comparing F4/80 positive cells and albumin positive cells**. A: Merged image showing green F4/80 positive cells and red microsphere positive cells. B: Same region as in 'A' photographed under ultraviolet optics to show DAPI positive nuclei. C: Merger of images shown in 'A' and 'B', demonstrating ovoid nuclear morphology of F4/80 and microsphere positive cells. D: Immunoreactivity for fluorescein labelled albumin. E: Same section as 'D', but ultraviolet optics reveal DAPI labelled nuclei. F: Merger of 'D' and 'E' demonstrating that albumin positive cells contain large round nuclei. Calibration bar in F = 50 μm for all images.

Figure 6 presents examples of the material analyzed for these studies, in this case taken from animals euthanized at P11. Figure 6A shows red microsphere containing and F4/80 immunoreactive cells. This same section is shown in Figure 6B under ultraviolet fluorescence optics to reveal the DAPI labelled cell nuclei, and the merger of all three fluorescence images is shown in Figure 6C. It can be seen that nuclei of the putative Kupffer cells have ovoid nuclei, in contrast to the large round nuclei that are seen more frequently in the tissue.

Figure 6D presents images from the adjacent section, processed for albumin immunoreactivity to identify the parenchymal hepatocytes. When this image is merged with an ultraviolet image showing the DAPI labelled nuclei (Figure 6E,F) it can be seen that the albumin positive cells contain the large round DAPI labelled nuclei.

Counts were made of F4/80 positive cells with clear DAPI labelled ovoid nuclei, and compared to counts from adjacent or neighboring liver sections of albumin positive cells with clear DAPI labelled large round nuclei; a ratio of hepatocytes to Kupffer cells was determined for each age. These metrics, summarized in Table 1 indicate no general trend in the number of F4/80 positive Kupffer cells, relative to the number of albumin positive cells, in the early postnatal period.

**Table 1:** Ratios of numbers of hepatocytes (H: albumin positive cells) to Kupffer cells (K: F4/80 positive cells).

Age (n)	Hn (d)	H nr/area	Kn (Lg d)	Kn (St d)	K nr/area	Ratio H:K
P3 (2)	10.3 (0.14)	29.7 (2.1)	9.5 (0.10)	4.3 (0.06)	6.3 (1.6)	4.7:1 (0.62)
P6-8 (4)	9.9 (0.15)	30.2 (3.2)	8.2 (0.17)	4.0 (0.10)	9.1 (2.1)	3.3:1 (0.27)
P10-11 (3)	9.6 (0.22)	28.6 (5.4)	8.6 (0.20)	4.0 (0.11)	9.1 (2.0)	3.6:1 (0.29)
P15-16 (3)	9.6 (0.19)	29.9 (2.9)	8.0 (0.25)	4.1 (0.10)	8.5 (1.4)	3.5:1 (0.29)
P20-21 (2)	9.4 (0.20)	31.7 (3.4)	8.0 (0.25)	4.1 (0.15)	8.0 (1.5)	3.9:1 (0.32)

Data include: Ages and numbers (n) of animals in each age; Diameter (d, in μm) of hepatocyte nuclei (Hn) and numbers of positive cells (H) in an area (nr/area) of 46,800 μm^2 ^(260 μm × 180 μm); Diameter (d, in μm) of Kupffer cell nuclei (Kn), both long axis (Lg d) and short axis (St d) and numbers of positive cells (K) in an area of 46,800 μm^2^; Ratios of numbers of hepatocytes (H)/numbers of Kupffer cells (K). Data are given as: mean (standard error).

## Discussion

### Technical considerations

Two techniques were employed to identify Kupffer cells in developing mice. Immunoreactivity for F4/80 was used in early studies to identify macrophages in mice [22] and since that time has been demonstrated to provide a valid marker of macrophages throughout the body and in a variety of species. In addition, administration of fluorescently labelled latex microspheres took advantage of the phagocytic activity of the Kupffer cells, and demonstrated the Kupffer cells engulfed the microspheres and led to the co-localization of microsphere labeling and F4/80 immunoreactivity.

Microspheres typically are administered intravascularly by injection into the tail vein. While this approach works well in adults, the small size of developing mouse pups clearly poses a challenge to making reliable tail vein injections. Although some investigators have provided instructions on intravenous injection (into scalp veins) in mouse pups as young as P5 [23], we were not able to achieve reliable injections at the younger ages. We were curious whether intraperitoneal injections might be effective. Comparison of aged matched controls revealed no differences in the distributions of microsphere labelling following intravenous vs. intraperitoneal injections, although the intravenous approach generally led to more intense labelling. This finding indicates that greater numbers of fluorescently labelled latex microspheres reached and were phagocytosed by Kupffer cells after IV injection as compared to IP injection. This result is not surprising in light of the requirement that with IP injections, the microspheres would need to first cross both the mesothelial lining of the visceral peritoneum and then cross either an endothelial barrier to enter the blood stream or a more permeable endothelial barrier to join the lymph; these steps may well reduce availability of the microspheres in reaching the Kupffer cells of the liver sinusoids. However, the similarity in patterns of labelling give support to the notion that intraperitoneal injection provides a valid approach for Kupffer cell labelling in younger pups. In support of this notion, we [24] found that peptide-containing liposomes target liver hepatocytes when administered either IV or IP in young postnatal mice. Further, a recent report [25] demonstrated that patterns of Evans Blue labelling were similar following IV and IP injections in mice.

When comparing the F4/80 labelling to the microsphere distribution it is evident that the size of the microsphere is important for determining their distribution pattern. The larger (0.2 μm) microspheres appear to be taken up within the liver primarily by the F4/80 positive Kupffer cells, while the smaller (0.02 μm) microspheres appear to be taken up not only by the Kupffer cells, but also by the CD-34 positive endothelial cells. Not all microspheres can be identified conclusively as being within specific cell types; some of the microspheres appear to be located extracellularly, perhaps adhering to the plasmalemma of either Kupffer or endothelial cells prior to being engulfed by those cells.

### Identifying Kupffer Cells

The types of cells that comprise the mouse liver are similar to those that have been described in other mammalian species. The most prominent cell type is the parenchymal hepatocyte [8-10,21]. Non-parenchymal cells include the phagocytic Kupffer cells [1-3,7,12-17,21], labelled with the F4/80 antibody [21,22], which in the adult mouse liver are approximately 35% of the number of hepatocytes, and also the Ito stellate cells [26-30], whose numbers are about 8-10% of the number of hepatocytes. As with any organ, endothelial cells form much of the lining of the sinusoidal capillaries. Although the thin squamous endothelial cells do not contribute a great deal to the volume of tissue in the liver, the number of endothelial nuclei in adult mouse liver is approximately 22% of all liver nuclei and approximately 40% of the number of hepatocytes [21].

Early studies demonstrated that Kupffer cells can be identified by their ability to phagocytose a variety of tracer substances, including carbon, India ink, or latex microspheres [12,15,21,26,31,32], and also by their immunoreactivity to the F4/80 antibody [21,22]. The use of latex microspheres of different diameters in the present study demonstrated that Kupffer cells could be labelled specifically with larger (0.2 μm) microspheres, while smaller microspheres (0.02 μm) labelled both Kupffer cells and endothelial cells, as has been demonstrated previously [12].

Previous investigations [6,7] have noted that Kupffer cells are more frequently encountered and also are larger in regions around the portal areas than around the central venules. The present data corroborate this finding in the developing mouse, although the regional differences in the developing mouse liver appear not as great as the regional differences reported for rat liver.

Liver endothelial cells are specialized, with the presence of fenestrations of approximately 100 to 140 nm diameter that appear aggregated into groups that form 'sieve plates' [1,3]. The very sparse nature of a basal lamina beneath the endothelial cells, along with the absence of diaphragmatic coverings of the fenestrations, allow for relatively free movement of small molecules between the capillary lumen and the space of Disse abutting the basolateral plasmalemmae of hepatocytes. Interestingly, neither the smaller (0.02 μm) nor the larger (0.2 μm) latex microspheres are detected in hepatocytes after intravascular injection, although they do appear to label endothelial cells. The 100-140 nm fenestrations of the liver endothelial cells are sufficiently large to allow movement of the smaller microspheres from the circulating blood into the space of Disse, and their absence from hepatocytes suggests that the microspheres either do not reach the space of Disse or are not taken up by the hepatocyte microvillous border within the space of Disse. Electron microscopic studies would be very useful in settling this issue.

### Development of Kupffer cells in postnatal mice

The early postnatal period (from P0 to approximately P21) is a time of active cellular differentiation and development. Counts of cells are difficult to make, because not only are cells migrating and proliferating, but also they are acquiring phenotypic markers that allow their identification. We attempted to gain quantitative estimates not of the absolute numbers of Kupffer cells in liver during the developmental period, but rather the numbers of Kupffer cells relative to numbers of hepatocytes. A conservative approach was taken, counting only those cells labelled by the appropriate immunoreactivity (F4/80 for Kupffer cells; albumin for hepatocytes) that also contained a DAPI labelled nucleus. Abercrombie's [33] method was used to reduce errors stemming from double counts of nuclei split between adjacent sections. Other systemic errors can influence the results, including estimates of sizes of nuclei with irregular shapes, such as those characteristic of Kupffer cells. The method of Abercrombie [33] is not as powerful as more modern stereological techniques, but was chosen because we did not have the sequential sections necessary for strict stereological approaches.

Numbers of Kupffer cells, relative to numbers of putative hepatocytes, appear low early in development, compared to the adult state [22]. This may seem surprising in light of the suggested phagocytic role for Kupffer cells during the early phase of hemotopoesis in the liver. Numbers of Kupffer cells of course relies upon the validity of F4/80 immunoreactivity. Whatever the function (currently not well understood) of the F4/80 antigen, it may have different distributions and antigenicity in the developing as compared with the mature liver. Previous studies [34,35] have demonstrated that Kupffer cells can be identified even in the fetal liver, by their phagocytic ability and expression of their F4/80 immunoeactivity. Further, hepatocytes can be identified by a variety of transcription factors and proteins, including albumin [35-37].

The spatial distributions of F4/80 positive cells and of the 0.2 μm diameter microsphere containing cells seen in developing mouse liver are similar to distributions of those same markers seen in the adult. Liver tissue collected from animals from 15 to 24 days of age appeared indistinguishable from that of adults, as regards the distribution and apparent intensity of F4/80 or microsphere labelling. Microsphere labelling was evident even at the youngest ages studied (P0 to P3), as was immunoreactivity to the F4/80 antibody and, as in the adult, these two markers were largely co-localized in the same cells. At the fine structural level [21], F4/80 immunoreactivity appears associated with the plasmalemmae of Kupffer cells. While the F4/80 antibody is commonly used as a marker for macrophages throughout the body, the cellular function of the antigen itself is not known.

Morphological differences are apparent between F4/80 positive cells taken from early postnatal liver tissue and those taken from mature animals. Mature Kupffer cells are morphologically complex, with extensive dendritic-like processes. In the early postnatal period, the dendritic processes appear less extensive, although longer and broader processes are common by P11. Whether these apparent morphological differences are due to real structural differences of the cells at different ages or due to differences in distribution of the F4/80 identified antigen is not clear at this time.

The finding that microsphere labelling of Kupffer cells in tissue from post-natal day 3 mice was similar to labelling in tissue from 12 week old mice indicates that the ability of Kupffer cells to recognize and engulf latex microspheres appears similar across ages. Of course, latex microspheres, while useful experimentally, are unlikely to be encountered in the natural life span of Kupffer cells from normal mice, and it may be that differences in recognition of different antigenic particles may be reflected in different rates of engulfing foreign particles as the animals age. The presence of phagocytically active Kupffer cells in these young animals supports the notion that those cells may be active in removing foreign antigens, including microbes, from the circulating blood. In addition, however, they may play a role in the removal of cell debris from the active process of hepatocyte formation and of hematopoiesis in the early postnatal liver. Future studies could include determining the age at which Kupffer cells first appear to be active participants in the immune system.

## Conclusions

Genetically engineered mice will play a very important role in future studies of liver function, and so it is vitally important to have baseline reference information on the cellular makeup of normal mouse liver. The present paper, using histological and immunocytochemical analyses, demonstrates that the population of Kupffer cells of the mouse liver is quite similar to that of other mammalian species, confirming and strengthening that the mouse presents a useful animal model for studies of Kupffer cell structure and function.

## Methods

### Materials

Chemical supplies were purchased from Sigma Aldrich (St. Louis MO) unless specified otherwise.

### Animals

All animal work was reviewed and approved by the University of California, Irvine Institutional Animal Care and Use Committee prior to conducting experiments, and all work was consistent with Federal guidelines. The ICR mice used in these experiments were purchased from Charles River (Wilmington CA) as pregnant dams or dams with litters of known age. Mice from newborns (postnatal day 0; P0) to P21 were kept with the dams in standard laboratory cages with nesting material. Pups were weaned at P21 and until 2 months of age were maintained in group cages and provided with standard laboratory mouse food and water ad libitum. All mice were housed in a vivarium with 12 h light and 12 h dark cycles.

### Tissue preparation

For studies of normal structure, mice were deeply anesthetized with sodium pentobarbital (50 mg/kg, IP). Mice were perfused through the heart with 5-10 ml room temperature saline, using a perfusion pump at a flow rate of 2-5 ml/min, to clear the vascular system of blood, then followed with cold 4% paraformaldehyde in sodium phosphate buffer (pH 7.4) for approximately 15 minutes.

The liver lobes were carefully removed, cut into 2-3 mm blocks, and fixed for an additional 1-18 hours before being placed in 30% sucrose for cryoprotection. Blocks of liver tissue were frozen in -20°C 2'methylbutane in preparation for sectioning with a cryostat. Frozen liver sections were cut on a Reichert-Jung 1800 cryostat at 10-12 μm; sections were mounted directly on Superfrost/Plus slides (Fisher Scientific, Pittsburgh PA), and air dried for 10-30 min before processing for immunocytochemistry.

### Latex microsphere injections

Mice were lightly anesthetized with Ketamine-xylazine (100 mg/kg Ketamine; 5 mg/kg xylazine; IP). Mice aged P16 and older received injections into the tail vein of 25-100 μl of a saline solution containing Fluorospheres (fluorescently labeled microspheres; 2.5%; Molecular Probes - Invitrogen, Carlsbad CA). Mice ages P0 to P16 received injections of 25-50 μl of the Fluorospheres in saline, IP, into the lower left quadrant of the peritoneal cavity. Microspheres of red fluorescence (excitation 580 nm; emission 605 nm) with mean diameters of either 0.02 μm or 0.2 μm (20 or 200 nm) were used, or of green fluorescence (excitation 505 nm; emission 515 nm) with a mean diameter of 0.03 μm. Fluorescent microspheres were injected either separately or mixed together as a cocktail composed of equal volumes of the stock suspensions. Following post-injection survival times of 15 min to 6 weeks, animals were deeply anesthetized with sodium pentobarbital and perfused through the heart as described above.

### Immunocytochemistry

Cryostat cut sections of liver were collected on Superfrost/Plus coated slides (Fisher Scientific, Philadelphia PA) and processed for immunocytochemistry. Slides with tissue sections were rinsed in Tris buffer three times and blocked for 1 hour in 3% normal goat serum (InVitrogen, Carlsbad CA). Primary antibodies were tested parametrically, in dilutions of Tris buffer in blocking solution, to determine the optimal antibody concentration to be used. The macrophage (Kupffer cell) antibody F4/80 (rat anti-F4/80 from Serotec, Raleigh NC) was used at 1:1000. The endothelial cell CD-34 antibody (mouse monoclonal antibody from Vector Labs; Burlingame CA) was used at 1:100. The albumin antibody (fluorescein isothiocyanate labelled goat anti-mouse albumin from Bethyl Labs, Montgomery TX) was used at 1:500. Sections were exposed to solutions containing primary antibodies at room temperature and in the dark, overnight (16-18 hr). The following day, slides were rinsed in Tris buffer three times. The sections then were incubated for 2 hours with Alexa 488 goat anti-rat IgG for the F4/80 procedure or Alexa 488 goat anti-mouse for the CD-34, (Invitrogen; Carlsbad CA; each at 1:1000). The slides for albumin did not require a secondary antibody, as the primary antibody was fluorescein labelled. The Alexa 488 fluorophore was excited at 495 nm and emitted fluorescence at 519 nm, and was viewed using a fluoroscein filter set. Following incubation, slides were rinsed with Tris buffer and coverslips were attached with Vectashield anti-fade fluorescent mounting medium with DAPI; DAPI served as a blue (ultraviolet) fluorescent stain for cell nuclei and was viewed with the ultraviolet fluorescence filter set.

### Image collection and processing

Slides were examined using a Nikon epifluorescence microscope equipped with rhodamine, fluorescein, and ultraviolet filter cubes. Digital images were captured using a Nikon DS 5M digital camera and imported into Adobe Photoshop. When creating photographic plates for illustrations, brightness and contrast were adjusted for uniformity within a plate; no other alterations of images were done.

Numbers of immunocytochemically identified cells were determined for neighboring pairs of 12 μm thick sections, one processed for F4/80 immunoreactivity and the other processed for albumin immunoreactivity. The sections were viewed with a 40× lens, in an area of 46,800 μm^2 ^(260 μm × 180 μm), and photographed using fluorescein and ultraviolet filter sets. At least three different areas in each section were photographed and analyzed. In some cases, the two images for each set, taken with fluorescein and ultraviolet filter settings, were merged and counts were made of immunoreactive cells containing DAPI stained nuclei. In other cases, the nuclei could be identified as blank (dark) round or ovoid structures in the centers of the immunoreactive cells. Diameters of DAPI stained nuclei were measured using the Nikon DS-5M software for two point distances, or from Photoshop images, using a reticule. The average number of positive cells and standard deviation for each animal was calculated, and the overall mean number of cells with standard errors was calculated for each cell type and age. The numbers of labelled cells (defined as an identifiable nucleus amid immunoreactivity) in each defined area (260 μm × 180 μm) was adjusted by the formula presented by Abercrombie [33]:

in which P is the calculated average number of nuclei per region, A is the crude count of number of nuclei of labeled cells per section, M is the tissue section thickness (12 μm), and L is the average diameter of nuclei. Counts of numbers of labeled cells did not differ between material with DAPI stained nuclei and unstained nuclei, so the data were combined.

## Competing interests

The authors declare that they have no competing interests.

## Authors' contributions

BGL did injections, tissue processing and immunocytochemistry, some of the photomicroscopy, and contributed to writing the manuscript. MST did tissue processing and some of the photomicroscopy. JLB did tissue processing and development of the immunocytochemistry methods. KJL participated in the design of the study and analysis of the results. RTR participated in the design of the study, performed some of the injections and perfusions, did photomicroscopy and image preparation, and contributed to writing the manuscript. All authors read, contributed to, and approved the final manuscript.
